# Preclinical evaluation of TIGIT as a target to enhance efficacy and mitigate T cell exhaustion in multiple myeloma following BCMA-CAR-T therapy

**DOI:** 10.1038/s41419-025-08203-w

**Published:** 2025-12-19

**Authors:** Yuan Xia, Jianfeng Zhu, Rui Guo, Xuxing Shen, Min Shi, Na Shen, Lei Fan, Lijuan Chen

**Affiliations:** 1 Department of Hematology, Jiangsu Province Hospital, The First Affiliated Hospital of Nanjing Medical University, Nanjing, China; 2 Department of Hematology, The Affiliated Taizhou People’s Hospital of Nanjing Medical University, Taizhou School of Clinical Medicine, Nanjing Medical University, Taizhou, China

**Keywords:** Immunosuppression, Myeloma

## Abstract

Chimeric antigen receptor T-cell (CAR-T) therapy targeting B-cell maturation antigen (BCMA) has achieved notable efficacy in relapsed/refractory multiple myeloma (RRMM), yet most patients eventually relapse, highlighting the need to elucidate mechanisms of resistance. In this study, we investigated the role of TIGIT in resistance to BCMA-CAR-T therapy. Tumor-infiltrating T cells from RRMM patients receiving BCMA-CAR-T therapy were analyzed by single-cell RNA sequencing, and immune checkpoint expression was further assessed by flow cytometry. Functional assays, including luciferase-based cytotoxicity, CD107a degranulation, CFSE proliferation, and CD45RO/CD62L phenotyping, were performed to investigate the effects of TIGIT inhibition through knockout or anti-TIGIT monoclonal antibodies (mAbs). In a humanized immune cell-reconstituted mouse model, TIGIT blockade was further evaluated for its effects on tumor growth and T cell exhaustion. T cells from patients with early relapse exhibited higher TIGIT expression than those from patients with durable responses, and elevated TIGIT levels were correlated with increased tumor burden and poor prognosis. While TIGIT blockade exerted limited effects on CAR-T function in vitro, it markedly enhanced CAR-T proliferation, mitigated T cell exhaustion, and improved antitumor efficacy in vivo, particularly when mediated by anti-TIGIT mAbs. Transcriptomic profiling further revealed that TIGIT modulates CAR-T activity by regulating cytokines and chemokines pathways and T cell activation, findings that were preliminarily validated by functional assays. Collectively, these findings identify TIGIT as a critical regulator of resistance to BCMA-CAR-T therapy and highlight TIGIT blockade as a promising strategy to enhance CAR-T efficacy and overcome relapse in multiple myeloma.

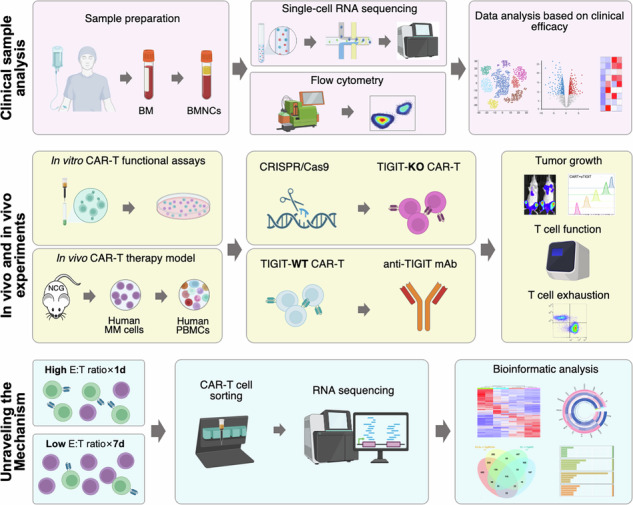

## Introduction

Multiple myeloma (MM) remains an incurable disease. Chimeric antigen receptor T-cell (CAR-T) therapy targeting B-cell maturation antigen (BCMA) has demonstrated remarkable efficacy in relapsed/refractory MM (RRMM). In the clinical trial of BCMA-CAR-T therapy (LCAR-B38M; NCT03090659) in which we participated, the overall response rate reached 87.8%, with a complete remission rate of 73% and a median progression-free survival (PFS) of 18 months over a follow-up period of 47.8 months [1]. Similarly, our systematic review of 22 BCMA-CAR-T studies for RRMM reported a median PFS of 14.0 months and a median overall survival (OS) of 24.0 months [2]. Thus, despite the encouraging response rates, relapse and resistance remain major challenges following BCMA-CAR-T therapy.

The mechanisms underlying relapse and resistance following CAR-T therapy remain incompletely understood. Current hypotheses implicate : (1) loss or downregulation of target antigens on MM cells, enabling immune escape; (2) exhaustion of CAR-T cells in vivo, impairing persistence and cytotoxicity; and (3) immunosuppressive tumor microenvironment, in which T cell exhaustion constitutes a major inhibitory component [3, 4]. T cell exhaustion is characterized by progressive loss of effector functions and memory properties under chronic antigen stimulation, as occurs in persistent infections or malignancies. In cancer, exhausted T cells fail to eliminate tumor cells, thereby promoting disease progression [5]. In B-cell leukemia, exhausted T cells have been shown to arise from CD8^+^ effector memory or terminal effector subsets, with high expression of immune checkpoints such as TIGIT, PD-1, and LAG-3 [6]. The upregulation of these immune checkpoints compromises T cell receptor (TCR) signaling, thereby reducing proliferative capacity and cytokine secretion [7].

Our previous single-cell RNA sequencing of bone marrow and peripheral blood samples from RRMM patients treated with BCMA-CAR-T revealed that patients who relapsed within one year exhibit diminished cytotoxicity and enhanced T cell exhaustion compared with patients exhibiting durable responses. This effect was most pronounced in CD8^+^ effector T cells. Moreover, relapsed patients show elevated expression of immune checkpoint molecules in both bone marrow and peripheral T cells, indicating that T cell dysfunction is a critical determinant of CAR-T resistance in MM [8]. Immune checkpoints are now established markers of T cell exhaustion, and their inhibition offers a potential strategy to restore T cell function. However, the dominant immune checkpoints vary across tumor types, which may partly explain the variable clinical efficacy of checkpoint inhibitors across malignancies. For example, CTLA-4 and PD-1/PD-L1 inhibitors have demonstrated substantial benefit in certain solid tumors and lymphomas [9], and combination strategies with CAR-T therapy are being explored. PD-1 blockade partially restores CAR-T function and augments the activity of CD19- and CD30-CAR-T cells [10–13]. Nevertheless, PD-1 inhibition has demonstrated limited efficacy in MM [14, 15], suggesting that other immune checkpoints play a more prominent role in this context. Indeed, a recent study investigated the role of TIGIT, a novel inhibitory receptor, in BCMA-CAR-T therapy using multiple blockade strategies, although no significant survival improvement was observed in vivo [16].

In this study, we investigate the contribution of TIGIT to BCMA-CAR-T resistance in MM and identify TIGIT as a potential therapeutic target to overcome CAR-T dysfunction. Using a humanized mouse model that better recapitulates the human immune microenvironment, we further evaluated the functional impact of TIGIT blockade in vivo. These findings provide a translational framework for developing novel checkpoint-targeting strategies to enhance the efficacy of BCMA-CAR-T therapy.

## Materials and methods

### Study population

Patients diagnosed with MM at the First Affiliated Hospital of Nanjing Medical University between January 2023 and October 2023 were enrolled. Diagnosis was established according to the revised International Myeloma Working Group (IMWG) criteria [17]. This study was conducted in accordance with the Declaration of Helsinki and approved by the Institutional Review Boards of the First Affiliated Hospital of Nanjing Medical University Ethics Committee (No. 2023-SR-011). Informed consent was obtained from all participants prior to enrollment.

### Cell lines and culture

Human RPMI-8226, U266, MM.1S, and 293T cell lines were purchased from American Type Culture Collection (ATCC, USA). RPMI-8226, U266 and MM.1S cells were cultured in RPMI-1640 medium (Gibco, USA) supplemented with 10% fetal bovine serum (FBS; Gibco, USA), while 293T cells were cultured in Dulbecco’s Modified Eagle Medium (DMEM; Gibco, USA) with 10% FBS. All cells were incubated at 37 °C in 5% CO_2_. Tumor cell lines stably expressing firefly-luciferase (ffluc) were generated via lentiviral transduction and maintained in medium containing 1 µg/mL puromycin.

### Generation of BCMA-CAR-T cells

The anti-BCMA CAR vector, kindly provided by Dr. Guang Hu from Iaso Biotherapeutics Co. Ltd. (Nanjing, China), comprised an anti-BCMA scFv linked to the CD8α hinge/transmembrane region, followed by 4-1BB and CD3ζ signaling intracellular signaling domains. A truncated epidermal growth factor receptor (EGFRt) was incorporated via a T2A linker.

Lentivirus was generated by transient transfection of 293 T cells with the CAR plasmid (anti-BCMA CAR vector) and packaging plasmids pMD2.G and psPAX2 using Lipofectamine 3000 (Invitrogen, USA). Viral supernatants were collected 48 h post-transfection and concentrated by centrifugation at 27,000 g for 4 h at 4 °C.

Peripheral blood mononuclear cells (PBMCs) from healthy donors were isolated by density gradient centrifugation. CD3^+^ T cells were purified using CD3 MicroBeads (Miltenyi, Germany), activated with CD3/CD28 Dynabeads (Gibco, USA), and cultured in X-VIVO 15 medium (Lonza, USA) supplemented with 5% FBS (Gibco, USA), 100 U/mL interleukin (IL)-2 (SL Pharm, China), and 2 mM L-glutamine (Gibco, USA). Twenty-four hours after activation, cells were transduced with concentrated lentivirus. Transduction efficiency was analyzed by flow cytometric detection of EGFRt expression.

### Generation of TIGIT-knockout BCMA-CAR-T cells by CRISPR/Cas9

TIGIT-knockout (TigitKO) CAR-T cells were generated using CRISPR/Cas9. A single guide RNA (sgRNA) targeting TIGIT (5’-ATGTCACCTCTCCTCCACCACGG-3’; GenScript, China) was complexed with recombinant Cas9 nuclease (Kactus Biosystems, China) to form Cas9 ribonucleoproteins (RNPs). CAR-T cells were resuspended in electroporation buffer, transferred to electroporation cuvettes, and electroporated using a cell electroporator (Celetrix Biotechnologies, USA) at 570 V for 20 ms. Cells electroporated with Cas9 alone (without sgRNA) under the same conditions were used as controls, designated as electroporation control CAR-T cells (EC-CAR-T). Genomic DNA was extracted using the QIAamp DNA Mini Kit (Qiagen, Germany), and the TIGIT locus was amplified by PCR. DNA sequencing was performed (Geneseeq Technology Inc., China), and editing efficiency was analyzed with the Synthego ICE tool (https://ice.synthego.com).

### Generation of gene knockdown BCMA-CAR-T cells by siRNA

BCMA-CAR-T cells were harvested during logarithmic growth, resuspended in pre-cooled electroporation buffer, and mixed with siRNA at a final concentration of 1 µM. After gentle mixing, the cell-siRNA mixture was transferred into an electroporation cuvette. Electroporation was performed at 480 V for 5 ms. Scrambled siRNA served as a negative control. All siRNAs were synthesized by Tsingke Biotech (Beijing, China) and are listed in Supplementary Table 1. Forty-eight hours post-electroporation, total RNA was extracted, and the knockdown efficiency of the target gene was assessed by quantitative PCR (qPCR).

### Flow cytometry

Cells were stained with fluorochrome-conjugated antibodies (details of clones, fluorochromes, suppliers are provided in Supplementary Table 2) according to the manufacturer’s instructions. For peripheral blood or bone marrow sample, 2 × 10^6^ nucleated cells were incubated with antibodies for 15 min at room temperature, followed by red blood cell lysis (Solarbio, China). For cultured cells, 1 × 10^6^ cells were washed twice and stained similarly. Data were acquired on a MACS Quant Analyzer 10 (Miltenyi, Germany) and analyzed with FlowJo v10.0 (BD Biosciences, USA).

T-cell subsets were defined as follows: naïve T cells (T_N_, CD45RO^–^ CD62L^+^ CD95^–^), stem cell-like memory T cells (T_SCM_, CD45RO^–^ CD62L^+^ CD95^+^), central memory T cells (T_CM_, CD45RO^+^ CD62L^+^), effector memory T cells (T_EM_, CD45RO^+^ CD62L^–^), and terminally differentiated effector T cells (T_TE_, CD45RO^–^ CD62L^–^).

### CFSE proliferation assay

CAR-T cells were labeled with CellTrace Violet Dye (Invitrogen, USA) and co-cultured with MM cells. Proliferation was monitored every 24 h by flow cytometry using 405 nm excitation. Each in vitro experiment was performed in triplicate, and representative results are presented.

### CD107a degranulation assay

CAR-T cells were co-incubated with MM cells with anti-CD107a antibody (1:50) and monensin (BioLegend, USA) for 6 h. CD107a expression was measured by flow cytometry as a marker of degranulation.

### Cytolysis assays

Luciferase-expressing tumor (2 × 10^4^) cells were co-cultured with CAR-T cells or mock T cells in U-bottom 96-well plates at varying effector-to-target (E:T) ratios in triplicate. After 24 h, luminescence was measured using a Synergy H1 microplate reader (BioTek, USA) with the SteadyGlo Luciferase assay system (Promega, USA).

### Generation of CAR-T cells in different functional states

BCMA-CAR-T cells were co-cultured with MM cells as previously described [18], generating distinct functional states: activated CAR-T cells (E:T ratio of 2:1 for 1 day) and exhausted CAR-T cells (E:T ratio of 0.1:1 for 7 days). After co-culture, CD3^+^ cells were purified using CD3 MicroBeads.

### Quantitative polymerase chain reaction (qPCR)

Total RNA was extracted using Trizol reagent (Invitrogen, USA), and cDNA was synthesized using the HiScript III RT SuperMix kit (Vazyme, China). qPCR was performed using ChamQ SYBR qPCR Master Mix (Vazyme, China) on the StepOne Real-Time PCR System (Applied Biosystems, USA). GAPDH was used as a reference gene. Relative expression was calculated using the 2^–ΔΔCt^ method. Primers used in this study were listed in Supplementary Table 3 and were synthesized by GenScript (China).

### Immunohistochemistry (IHC)

Bone marrow biopsy specimens from MM patients (n = 8) and healthy donors (n = 4) were decalcified with EDTA and embedded in paraffin. Sections were deparaffinized, rehydrated, and treated with 3% H_2_O_2_. Following antigen retrieval and blocking, slides were incubated with PVR/CD155 rabbit mAb (Cell Signaling Technology, USA) overnight at 4 °C, then with the secondary antibodies and a streptavidin-peroxidase complex. Staining was developed with DAB, counterstained with hematoxylin, and visualized under a light microscope.

### Single-cell RNA sequencing (scRNA-seq)

Single-cell RNA sequencing was performed on bone marrow specimens from RRMM patients prior to BCMA-CAR-T cell infusion, as detailed in our previous study [8]. Briefly, specimens were preserved in GEXSCOPE Tissue Preservation Solution and sent to Singleron Lab (China) for processing. After washing and filtration through 40 μm strainers, cells were centrifuged, resuspended in PBS, and subjected to red blood cell lysis. The single-cell suspension was assessed using Trypan blue and loaded onto microfluidic devices at a concentration of 1 × 10^5^ cells/mL. scRNA-seq libraries were prepared using the GEXSCOPE^®^ protocol and sequenced on an Illumina NovaSeq 6000, generating 150 bp paired-end reads. Gene expression profiles were generated using CeleScope, with barcodes and Unique Molecular Identifiers (UMIs) extracted, corrected, and aligned to the GRCh38 transcriptome. Uniquely mapped reads were assigned to exons using FeatureCounts. The Seurat package was used for quality control, clustering, and dimensionality reduction, with filtering based on gene count, UMI count, mitochondrial content (>50%), and gene expression in fewer than five cells.

### Bulk RNA sequencing

RNA libraries were sequenced on the Illumina NovaSeq 6000 platform (LC Bio Technology Co., Ltd., China) according to standard protocols. In brief, total RNA was extracted using TRIzol reagent, followed by assessment of RNA quantity and purity using a NanoDrop ND-1000 spectrophotometer (NanoDrop, USA). RNA integrity was evaluated with the BioAnalyzer 2100 system (Agilent, USA). Poly(A)-containing mRNA was selectively captured using oligo(dT) magnetic beads (Dynabeads Oligo(dT), Thermo Fisher, USA) through two rounds of purification. The captured mRNA was fragmented and reverse transcribed into cDNA. The RNA-DNA hybrid was then converted into a double-stranded DNA, which was processed, screened, purified, and digested. PCR amplification was performed to construct strand-specific libraries with an average fragment size of 300 ± 50 bp. Finally, paired-end sequencing (PE150) was conducted on the Illumina NovaSeq 6000 platform. Sequencing data were filtered to obtain clean reads, and high-quality data were aligned to the reference genome (GRCh38) used in this study. Gene and transcript quantification for each sample was performed using StringTie. Sample quality control and data characteristics are summarized in Supplementary Table 4.

### Identification and enrichment of differentially expressed genes (DEGs)

The Seurat “FindMarkers” function was employed to identify DEGs, utilizing the Wilcoxon rank-sum test with default parameters. A gene was classified as a DEG if it was expressed in >10% of cells in each of the compared groups and exhibited an absolute log (fold change) >0.25. Adjusted P value was calculated using Bonferroni correction, with a significance threshold set at P<0.05. Differential gene expression was visualized using the R packages pheatmap and circlize.

To further investigate the biological functions and signaling pathways associated with the DEGs, we conducted Gene Ontology (GO) and Kyoto Encyclopedia of Genes and Genomes (KEGG) enrichment analyses using the R package clusterProfiler. Pathways with an adjusted P<0.05 were considered significantly enriched.

### Protein–protein interaction (PPI) analysis

PPI networks were constructed using the STRING (https://cn.string-db.org/) and visualized in R using the igraph and ggraph packages.

### Mouse xenotransplantation model

Female NOD/ShiLtJGpt-Prkdc^–/–^Il2rg^–/–^/Gpt (NCG) mice, 6 weeks old and weighing 19–21 g, were purchased from GemPharmatech Co., Ltd (China). All animal care and experimental procedures complied with the Guidelines for the Care and Use of Laboratory Animals of Nanjing Medical University, and were approved by Institutional Animal Care and Use Committee (IACUC) of Nanjing Medical University (No. 2201014). Anesthesia was induced by intravenous injection of pentobarbital sodium. For tissue collection, mice were euthanized by cervical dislocation following intravenous injection of pentobarbital sodium. At the experimental endpoint, humane euthanasia was performed using carbon dioxide inhalation.

On day –10, mice were intravenously injected with 2 × 10^6^ RPMI-8226-ffLuc cells via the tail vein. To establish immune reconstitution, 2 × 10^7^ healthy donor-derived PBMCs were administered intravenously on day –5. On day 0, mice were randomized into five groups and treated as follows: (a) TigitKO-CART group: mice received 2 × 10^6^ TigitKO BCMA-CAR-T cells on day 0. (b) EC-CART group: control group for (a); mice received CAR-T cells electroporated without Cas9-RNPs, retaining TIGIT expression. (c) CART + αTIGIT group: mice received 2 × 10^6^ BCMA-CAR-T cells on day 0, followed by intravenous administration of anti-TIGIT monoclonal antibody (mAb) (Ociperlimab, BeiGene) at 3 mg/kg every 5 days starting on day 1. (d) CART + IgG group: control group for (c); mice received isotype control IgG1 (BioXCell, USA) at 3 mg/kg on the same schedule. (e) PBS group: Mice received an equivalent volume of PBS via tail vein injection on day 0. All CAR-T cells were derived from the same donor as the PBMCs used for immune reconstitution. The PBS group consisted of five mice, while all other groups included ten mice each. Blinding was not performed during the animal experiments.

Body weight was recorded every 5 days. Tumor burden was assessed weekly by bioluminescence imaging using the IVIS Spectrum Imager system (PerkinElmer, USA), and signal intensity was quantified with Living Image software (PerkinElmer, USA). Peripheral blood was collected for flow cytometric analysis to assess CAR-T cell frequency and phenotype. CAR-T cells were enriched by incubating samples with biotin-conjugated anti-human EGFR antibody (BioLegend, USA) followed by anti-Biotin MicroBeads (Miltenyi, Germany), according to the manufacturer’s instructions. EGFR^+^ cells (CAR-T cells) were subsequently analyzed for TCF7 and LEF1 gene expression by qPCR.

### Statistical analysis

For normally distributed variables, statistical comparisons were performed using Student’s *t* test or one-way ANOVA. For non-normally distributed variables, the Mann–Whitney *U* test or Kruskal-Wallis test was applied. Linear correlation between variables was assessed using Pearson’s correlation coefficient (*r*). All analyses were conducted using SPSS v23.0 (IBM, USA) or GraphPad Prism v8.0 (GraphPad Software, USA). All tests were two-tailed, and *P* < 0.05 was considered statistically significant.

## Results

### Single-cell RNA sequencing links post-CAR-T relapse to host T cell function and TIGIT expression

To investigate the mechanisms underlying relapse following BCMA-CAR-T therapy, we performed scRNA-seq on bone marrow cells from three patients with RRMM collected prior to BCMA-CAR-T cell infusion. Patients were categorized into two groups based on clinical outcome: an early relapse (ER) group (n = 1), defined as disease progression within 1 year of infusion, and a durable response (DR) group (n = 2), characterized by a response lasting more than 1 year.

**Fig. 1:**
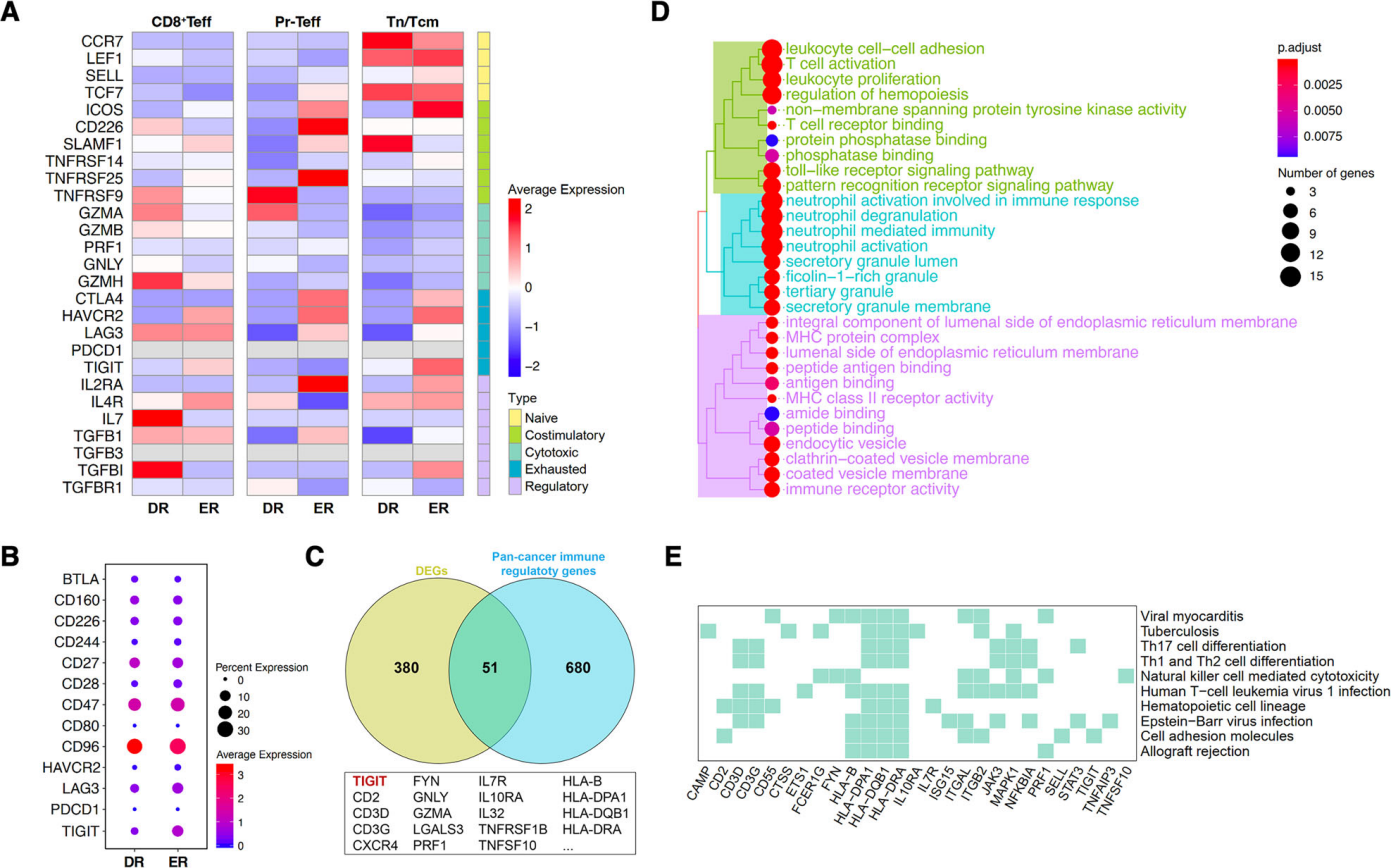
Single-cell RNA sequencing analysis of tumor-infiltrating T cells in MM patients with early relapse (ER) and durable response (DR) following BCMA-CAR-T therapy. **A** Expression of marker genes representing distinct functional states of T cell subsets in patients with different clinical responses. **B** Expression of immune checkpoints on T cells from patients with different response. **C** Venn diagram illustrating showing the overlap between DEGs in patients with different response and a pan-cancer immune regulatory gene profile. **D** Treeplot of GO enrichment analysis for the intersecting genes. **E** Heatplot of KEGG enrichment for the intersecting genes.

scRNA-seq analysis of immune checkpoints within tumor-infiltrating T cells revealed significantly higher TIGIT expression in T cells from the ER patient compared to DR patients (P < 0.05). This elevated expression was particularly prominent in CD8^+^ effector T cells (T_eff_) and naïve/central memory T cells (T_n_/T_cm_) (Fig. 1A, B). Furthermore, an analysis utilizing a pan-cancer immune regulatory gene profile [19] identified a distinct differential gene expression pattern related to tumor immune regulation between the DR and ER patients (Fig. 1C, Supplementary Table 5). Gene enrichment analysis indicated that these DEGs were mainly involved in pathways related to T cell activation, proliferation, differentiation, and neutrophil regulation (Fig. 1D, E). These findings suggest that dysregulation of tumor-infiltrating T cells, particularly the immune checkpoint TIGIT, may contribute to disease relapse following CAR-T therapy.

### TIGIT expression in tumor-infiltrating T cells correlates with MM progression

**Fig. 2:**
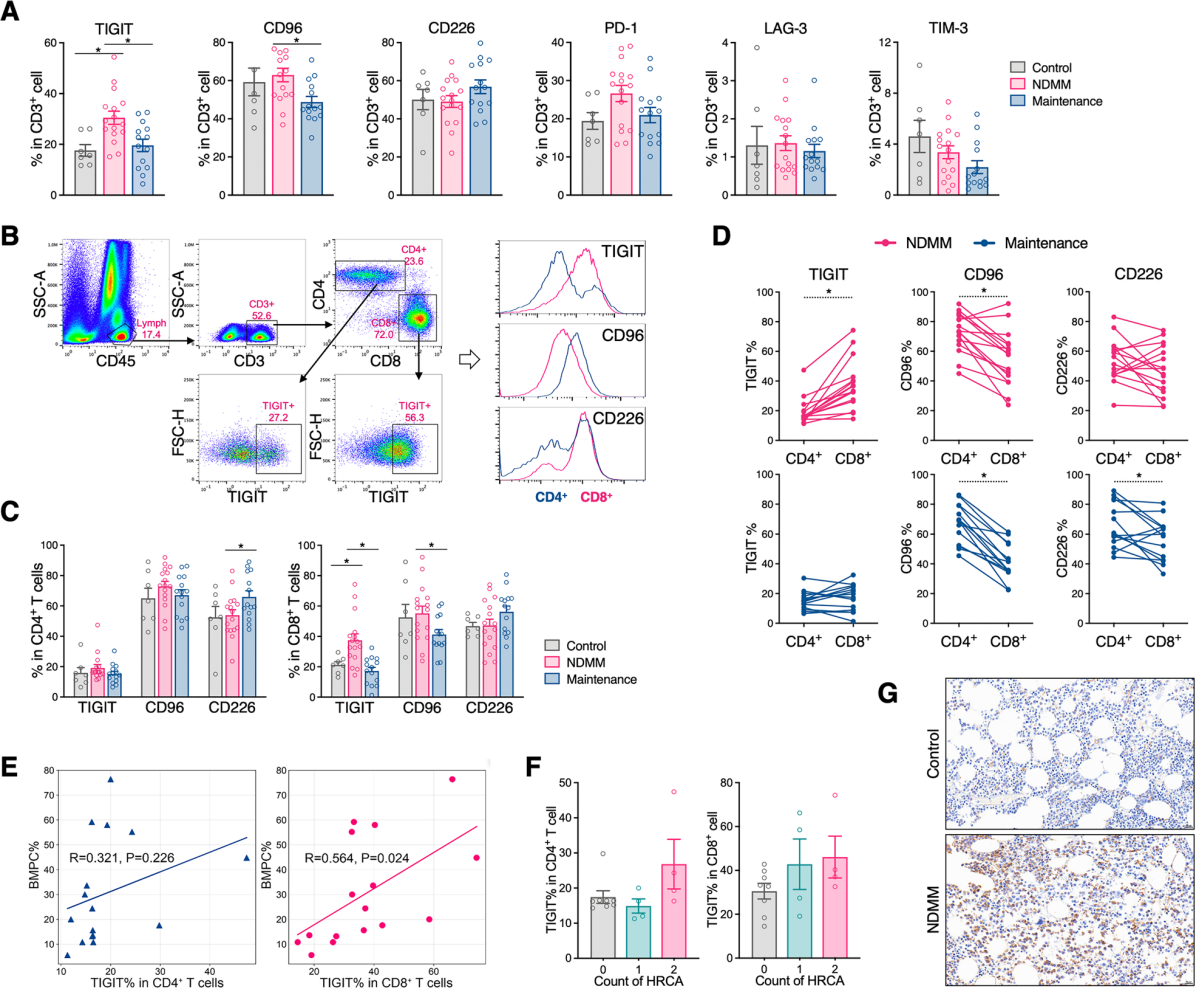
Immune checkpoint expression in bone marrow tumor-infiltrating T cells of MM patients in relation to clinical features. **A** Expression levels of immune checkpoints in bone marrow T cells from healthy donors (Control, n = 7), newly diagnosed MM patients (NDMM n = 16), and MM patients in remission (Maintenance, n = 14). **B** Flow cytometry gating strategy for analyzing TIGIT-axis molecules in CD4^+^ and CD8^+^ T cells. **C** Expression of TIGIT-axis molecules in CD4^+^ (left) and CD8^+^ (right) T cells from MM patients. **D** Paired comparison of TIGIT-axis molecule expression in CD4^+^ (upper) and CD8^+^ (lower) T cells from the same patient. **E** Correlation between TIGIT expression in bone marrow CD4^+^ (left) and CD8^+^ (right)T cells and the proportion of bone marrow plasma cell (BMPC). **F** Association between TIGIT expression in bone marrow CD4^+^ (left) and CD8^+^ (right) T cells and the number of high-risk cytogenetic abnormalities (HRCA). No HRCA, n = 8; one HRCA, n = 4; two HRCA, n = 4. **G** Immunohistochemical detection of CD155 expression in bone marrow biopsy specimens from healthy donors and NDMM patients (Data are presented as mean ± SEM; ^*^P < 0.05).

Given the critical role of immune checkpoints in MM, we used flow cytometry to examine the expression of key immune checkpoint molecules, with a focus on the TIGIT axis. We analyzed tumor-infiltrating T cells from bone marrow samples of healthy donors (Control, n = 7), newly diagnosed MM (NDMM, n = 16), and patients in remission (Maintenance, n = 14). TIGIT expression was significantly elevated in NDMM patients compared to both healthy controls and patients in remission (Fig. 2A). Sub-analysis revealed this increase was specific to CD8^+^ T cells in NDMM patients, with no similar elevation observed in CD4^+^ T cell populations (Fig. 2B, C). Interestingly, while elevated TIGIT expression was restricted to CD8^+^ T cells in NDMM patients, no such differences were detected in T cells from patients in remission (Fig. 2D). We also observed that CD96 expression was predominantly found on CD4^+^ T cells from MM patients.

Further analysis revealed a significant positive correlation between the proportion of bone marrow plasma cells (BMPC) and TIGIT expression in CD8^+^ T cells (P = 0.024), a correlation not found in CD4^+^ T cells (Fig. 2E). Additionally, patients with high-risk cytogenetic abnormalities (HRCA), which predict poorer prognosis, exhibited higher TIGIT expression on CD8^+^ T cells (Fig. 2F). Collectively, these findings suggest that the TIGIT axis, and particularly TIGIT itself, may contribute to MM progression through its predominant impact on CD8^+^ T cell function.

Given that the function of TIGIT is often mediated through interactions with ligands, we also examined the expression levels of CD155, a major ligand of TIGIT, in both MM patients and healthy donors. IHC analysis demonstrated a diffusely elevated expression of CD155 within the bone marrow microenvironment of MM patients (Fig. 2G).

### Exposure of BCMA-CAR-T cells to MM cells induces high TIGIT expression and a phenotype of exhaustion in vitro

**Fig. 3:**
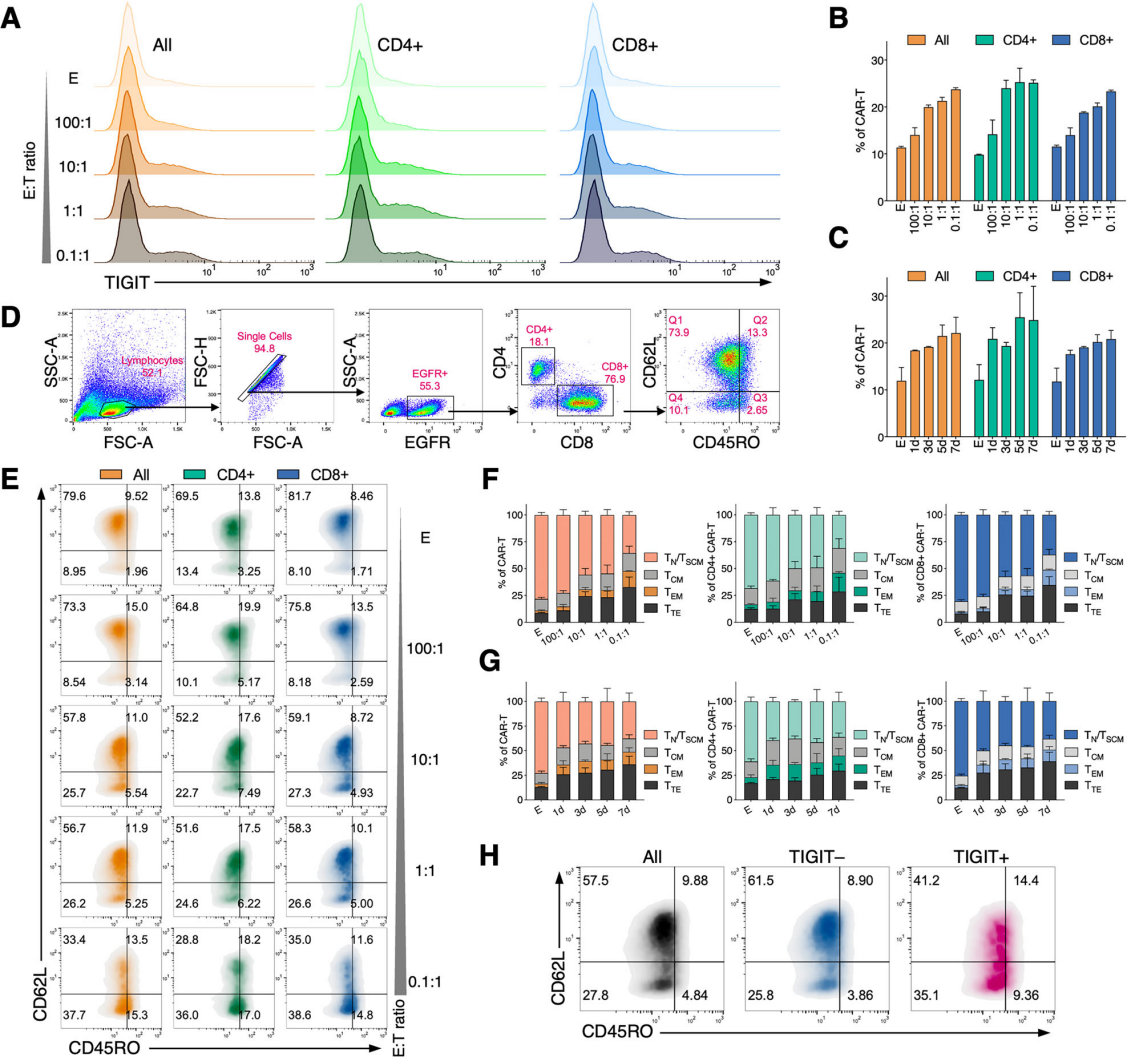
TIGIT expression and phenotypic characteristics of BCMA-CAR-T cells following antigen activation. **A** Representative histograms showing TIGIT expression on total CAR-T cells (left), CD4^+^ CAR-T cells (middle), and CD8^+^ CAR-T cells (right) at different antigen concentrations. **B** TIGIT expression levels on CAR-T cells under varying antigen concentrations. **C** TIGIT expression levels on CAR-T cells after co-culture with antigen over time. **D** Flow cytometric gating strategy for analyzing CAR-T cells phenotypes: naïve and stem cell-like memory (T_N_/T_SCM_; (CD45RO^–^ CD62L + ), central memory (T_CM_; CD45RO^+^ CD62L^+^), effector memory (T_EM_; CD45RO^+^ CD62L^–^), and terminally differentiated effector (T_TE_; CD45RO^–^ CD62L^–^). **E** Representative density plots showing CAR-T cell phenotypes after co-culture with target cells at different antigen concentrations.(left: total CAR-T cells; middle: CD4^+^ CAR-T cells; right: CD8^+^ CAR-T cells). **F** Quantification of CAR-T cell phenotypes after co-culture with target cells at varying antigen concentrations (left: total CAR-T cells; middle: CD4 + CAR-T cells; right: CD8 + CAR-T cells). **G** Temporal changes of CAR-T cell phenotypes after co-culture with MM cells. **H** Comparison of phenotypes between TIGIT^+^ and TIGIT^–^ CAR-T cells (Data are presented as mean ± SEM).

To investigate the effect of the tumor microenvironment on CAR-T cells, we co-cultured BCMA-CAR-T cells with MM cells at various E:T ratios. Flow cytometry analysis after 1 day showed a dose-dependent upregulation of TIGIT expression on CAR-T cells, with higher concentrations of target cells corresponding to increased TIGIT level (Fig. 3A, B). A similar trend was observed when CAR-T cells were co-cultured with MM cells at a fixed E:T ratio of 1:1, with TIGIT expression progressively increasing over time (Fig. 3C). This demonstrates that MM cells can induce TIGIT expression on CAR-T cells in a dose- and time-dependent manner.

We also assessed the phenotypic changes of CAR-T cells following co-culture. Using flow cytometry to analyze surface expression of CD45RO and CD62L (gating strategy shown in Fig. 3D), we found that higher target cell concentrations led to a decreased proportion of memory phenotype cells and a boosted population of terminally differentiated effector cells (Fig. 3E, F). Prolonged co-culture time had a similar effect, promoting CAR-T cell differentiation toward a terminal effector stage. These results suggest that elevated tumor antigen concentration and extended exposure time are likely to promote CAR-T cell differentiation toward a state of exhaustion.

### Generation of TigitKO BCMA-CAR-T cells using CRISPR/Cas9

**Fig. 4:**
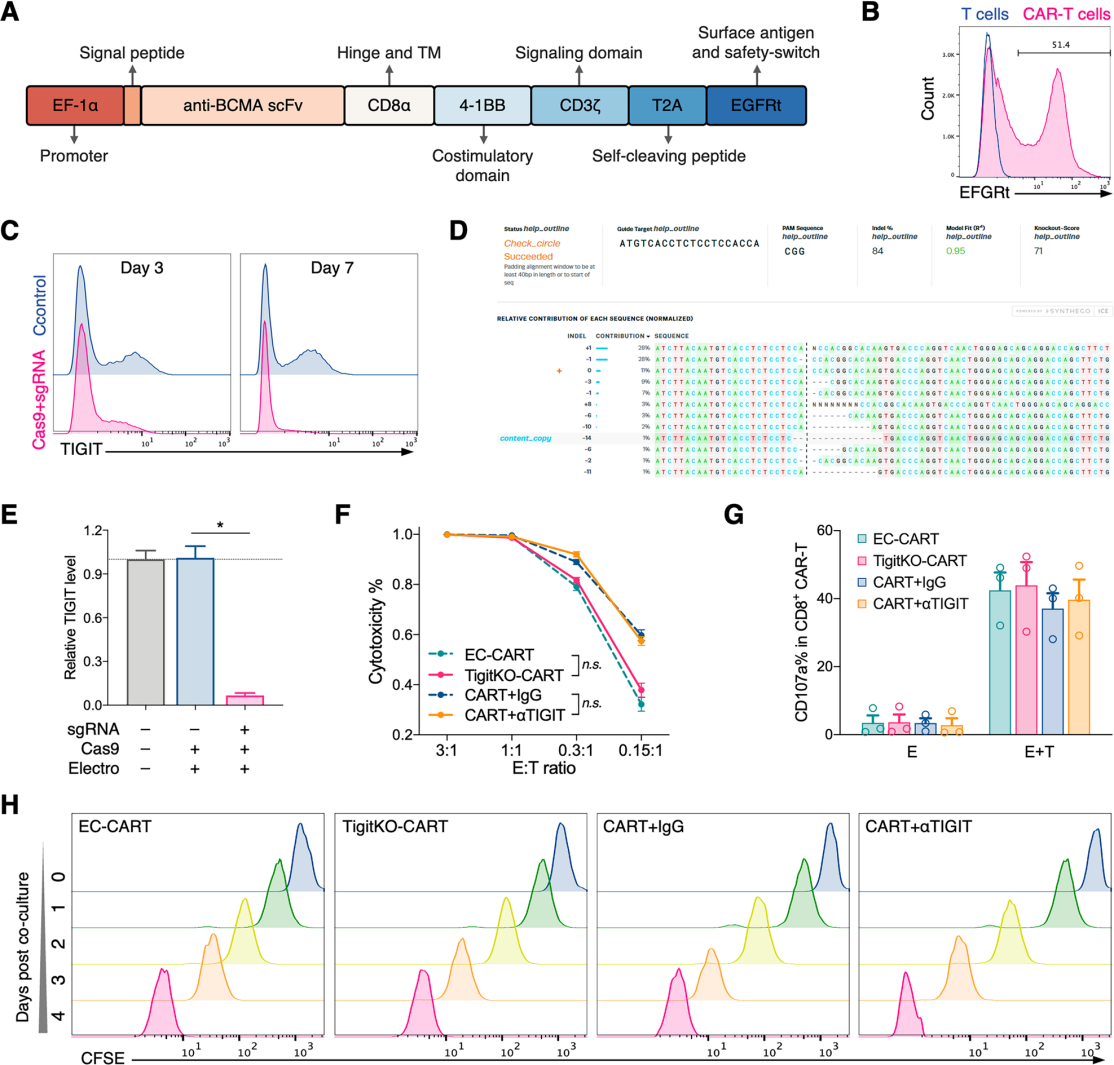
Generation and functional characterization of TIGIT-knockout BCMA-CAR-T cells in vitro. **A** Schematic diagram of the anti-BCMA CAR construct. **B** Flow cytometry analysis of CAR-T cell transduction efficiency. **C** Flow cytometry assessment of TIGIT expression on CAR-T cells at 3 and 7 days after CRISPR/Cas9 editing. **D** Validation of TIGIT gene editing efficiency by DNA sequencing. **E** qPCR analysis of TIGIT mRNA expression following CRISPR/Cas9 editing. **F** Effect of TIGIT knockout on CAR-T cell degranulation assessed by CD107a assay. **G** Effect of TIGIT knockout on CAR-T cytotoxicity measured by cytolysis assays. **E** (effector cells) represent CAR-T cells, T (target cells) represent MM cells. **H** Effect of TIGIT knockout on CAR-T proliferation of CAR-T cells measured by CFSE assay (Data are presented as mean ± SEM; ^*^P < 0.05).

To functionally investigate the role of TIGIT in CAR-T cells, we generated TigitKO BCMA-CAR-T cells using CRISPR/Cas9. Lentiviral transduction was first employed to express the BCMA-CAR (Fig. 4A, B), followed by TIGIT gene editing via electroporation. Flow cytometry confirmed a significant decrease in surface TIGIT protein on edited CAR-T cells (Fig. 4C). DNA sequencing verified a gene editing efficiency of 71% (Fig. 4D), and qPCR analysis showed a significant reduction in TIGIT mRNA levels (Fig. 4E). This validated our approach for generating TigitKO CAR-T cells for further study.

### In vitro TIGIT blockade has minimal impact on BCMA-CAR-T cell function

We employed two distinct strategies to block TIGIT on CAR-T cells—TIGIT knockout and treatment with a TIGIT mAb—to clarify TIGIT’s effects on CAR-T cell function in vitro. Using a luciferase assay to measure cytotoxicity and flow cytometry to measure CD107a expression as a proxy for degranulation, we found that neither TIGIT blockade method significantly affected the cytotoxic activity (Fig. 4F) or degranulation (Fig. 4G) of CAR-T cells upon exposure to target antigens. A CFSE assay evaluating proliferation revealed that CAR-T cells treated with TIGIT mAb exhibited a slightly enhanced proliferation signal compared to the control group (Fig. 4H). In contrast, TIGIT knockout did not significantly impact in vitro CAR-T cell proliferation. These results suggest that in a controlled in vitro setting, TIGIT blockade has a limited effect on the short-term function of CAR-T cells.

### In vivo TIGIT blockade reduces T cell exhaustion and enhances anti-tumor effects

**Fig. 5:**
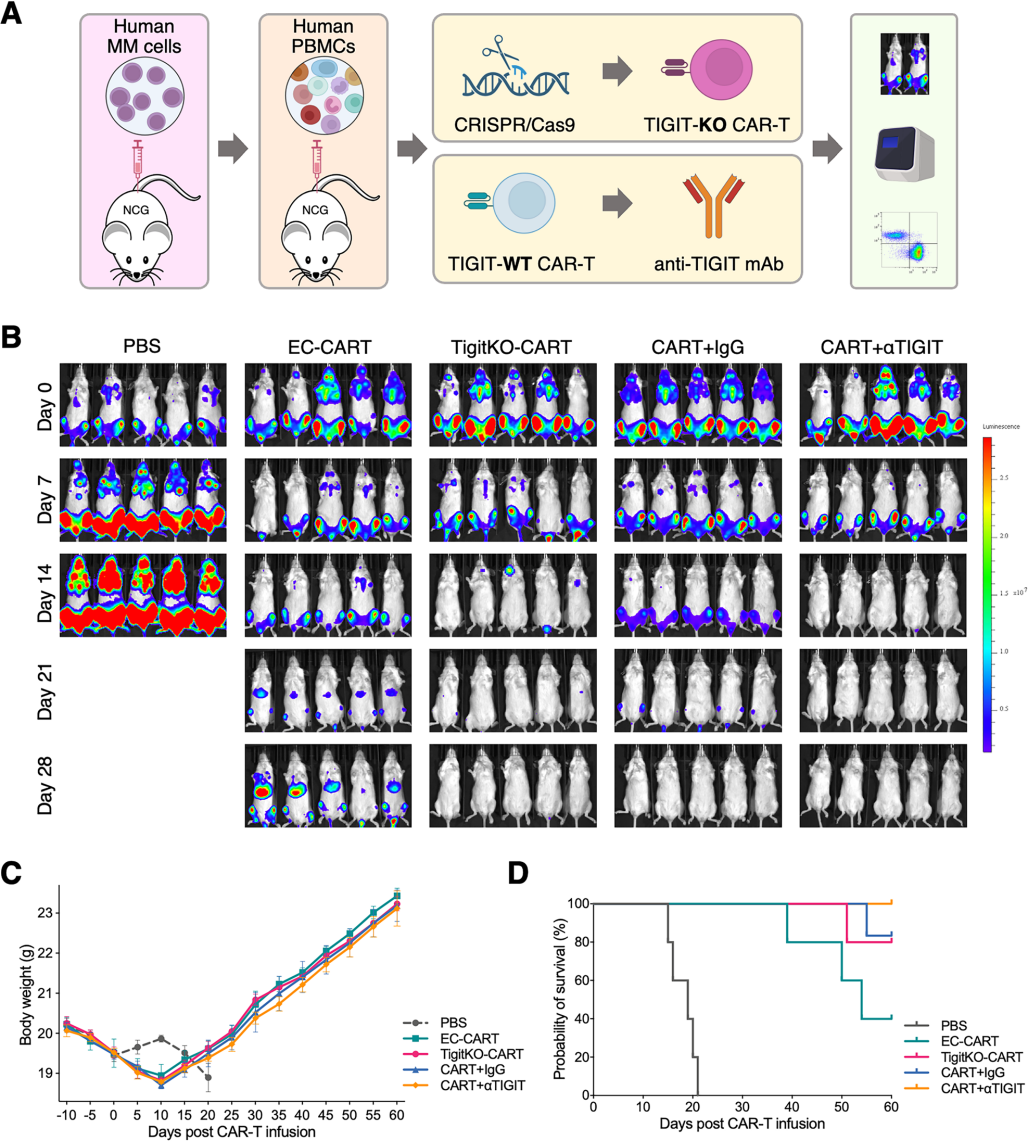
In vivo TIGIT blockade enhances the antitumor efficacy of BCMA-CAR-T therapy in MM mouse models. **A** Schematic diagram of the animal model and experimental workflow. **B** Bioluminescence images of tumor burden at different time points after CAR-T infusion (day 0 indicates the infusion day). **C** Changes in mouse body weight over time. **D** Kaplan–Meier survival analysis of mice up to 60 days after CAR-T infusion.

To better model the clinical setting, we conducted an in vivo study using humanized NSG mice reconstituted with human PBMCs, followed by infusion of CAR-T cells from the same donor. We again used both TIGIT knockout and TIGIT mAb blockade methods to investigate their effects on CAR-T cell functionality in vivo (Fig. 5A). In vivo imaging demonstrated that the CART + αTIGIT group achieved superior and more rapid tumor clearance compared to the control (CART+IgG) group. Similarly, TigitKO CAR-T cells also showed stronger and faster anti-tumor activity than their respective controls. Interestingly, the anti-tumor effect in the CART + αTIGIT group exceeded that of the TigitKO CAR-T group (Fig. 5B). No significant differences in body weight were observed among any CAR-T treatment groups (Fig. 5C). Survival analysis indicated that the CART + αTIGIT group had the best outcome, with 100% survival at the 60-day endpoint. In contrast, the EC-CART group exhibited the poorest survival, consistent with the observed tumor burden (Fig. 5D).

**Fig. 6:**
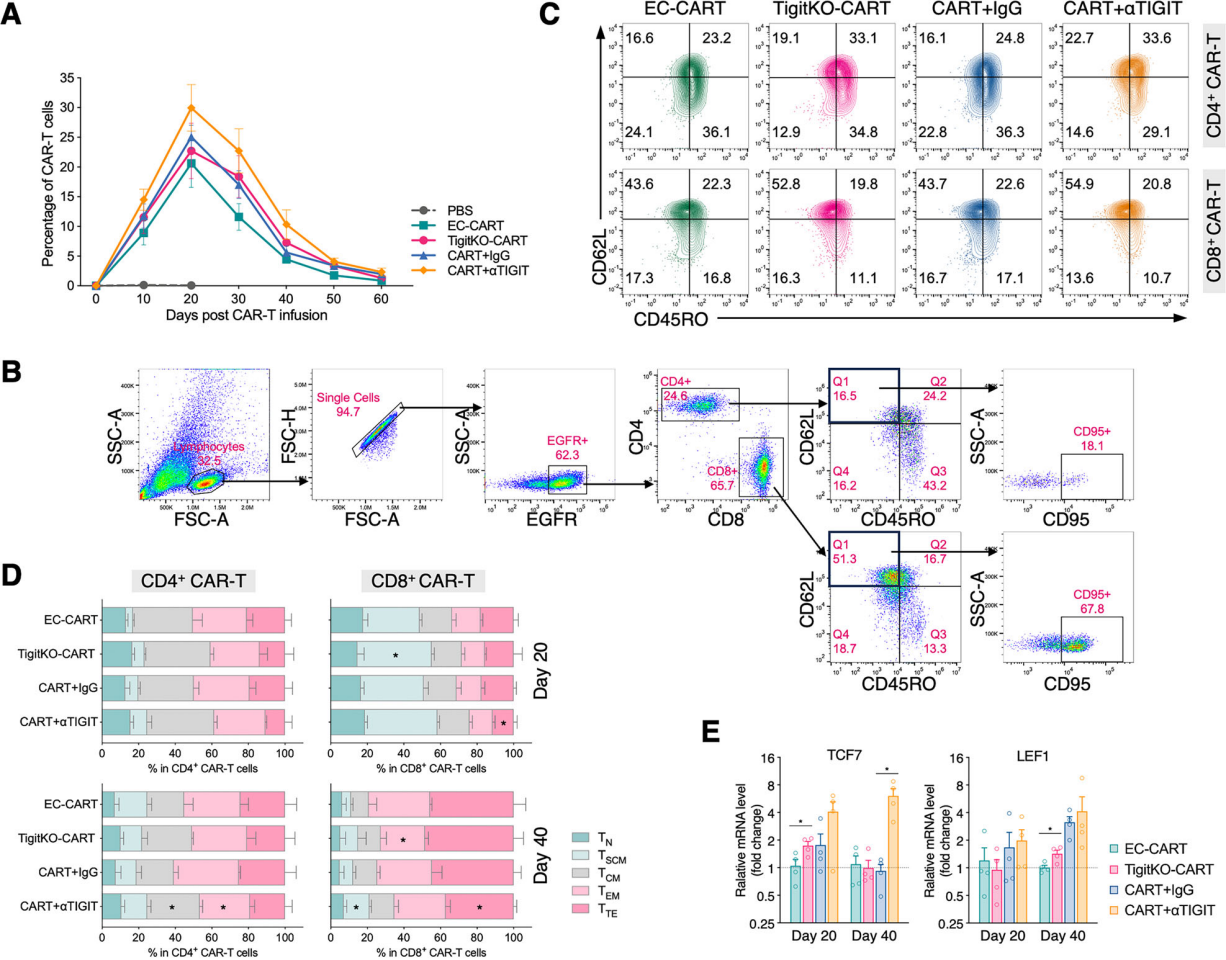
In vivo effects of TIGIT blockade on the expansion and phenotype of BCMA-CAR-T cell. **A** Percentage of CAR-T cells in peripheral blood up to 60 days after infusion. **B** Gating strategy used in flow cytometry to analyze CAR-T cells phenotypes in mice. **C** Representative plots showing CAR-T cell phenotype in treated mice on day 20. **D** Comparison of CAR-T cell phenotypes among different treatment groups on days 20 and 40 post-infusion (* indicates a statistically significant difference compared with the corresponding control group). **E** qPCR analysis of TCF7 and LEF1 expression levels in CAR-T cells on day 20 post-infusion (Data are presented as mean ± SEM; ^*^P < 0.05).

To quantify CAR-T cell expansion, peripheral blood was drawn from the murine buccal vein for flow cytometric analysis. CAR-T cell levels peaked around day 20 after infusion, then declined, reaching ~1.6% at day 60 (minimum 0.45%). TIGIT blockade enhanced the expansion potential of CAR-T cells, with the TIGIT mAb group showing the most pronounced expansion among all groups (Fig. 6A). Flow cytometric analysis at both the expansion peak (day 20) and a later time point (day 40) showed that CAR-T cells from TIGIT-blockaded mice retained early memory phenotypes more effectively and expressed fewer terminal differentiation markers compared to those in control groups (Fig. 6B–D). Further qPCR analysis revealed that TIGIT blockade led to elevated expression levels of TCF7 and LEF1 in CAR-T cells, with a particularly pronounced upregulation of TCF7 (Fig. 6E).

### RNA-seq reveals significant impact of TIGIT on cytokine and chemokine pathways

**Fig. 7:**
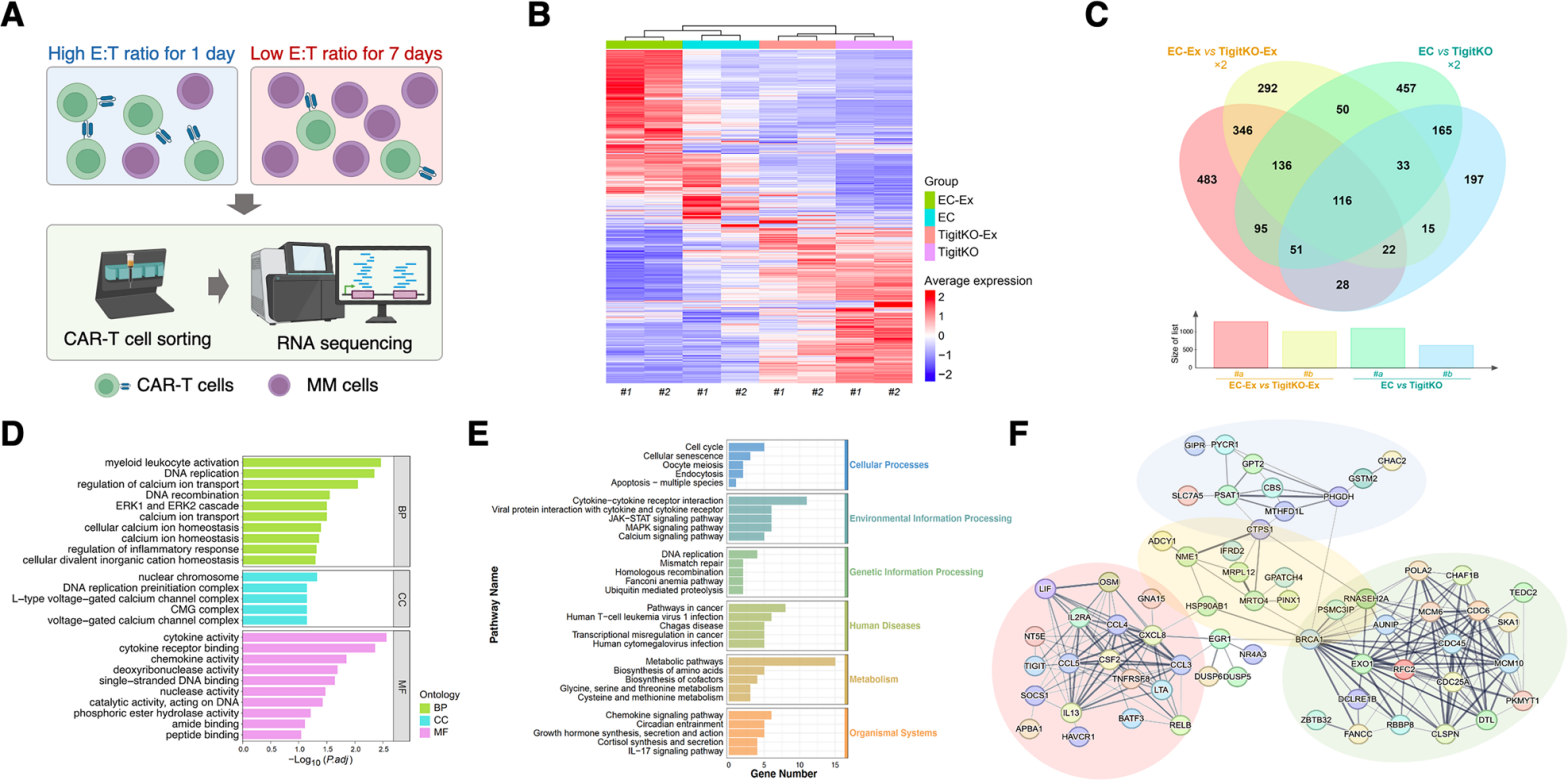
RNA-seq analysis of the transcriptional impact of TIGIT knockout in CAR-T cells. **A** Experimental design of RNA-seq: CAR-T cells were co-cultured with MM cells at a high E:T ratio (2:1) for 1 day (activated CAR-T cells) or at a low E:T ratio (0.1:1) for 7 days (exhausted CAR-T cells). CAR-T cells were isolated by magnetic sorting for RNA-seq. **B** Heatmap showing clustering of gene expression across groups. EC: activated EC-CAR-T; EC-Ex: exhausted EC-CAR-T; TigitKO: activated TIGIT-knockout CAR-T cells; TigitKO-Ex: exhausted TIGIT-knockout CAR-T cells. **C** Venn diagram showing DEGs across groups. **D** GO enrichment analysis of the 116 intersecting DEGs. **E** KEGG enrichment analysis of the 116 intersecting DEGs. **F** PPI network of the 116 intersecting DEGs, with the cluster containing TIGIT highlighted in light red (lower left).

To elucidate the molecular mechanisms underlying the in vivo observations, we performed RNA-seq on BCMA-CAR-T cells. To induce distinct activation and exhaustion states, CAR-T cells were co-cultured with MM cells at high or low E:T ratios for 1 or 7 days. CAR-T cells were then isolated using immunomagnetic bead for subsequent RNA-seq (Fig. 7A). Two biological replicates were prepared for each condition, labeled as #1 and #2. Pairwise analysis of TigitKO CAR-T cells and the control EC-CAR-T cells identified 365 DEGs in the activated state and 620 in the exhausted state, with 116 genes common to both conditions (Figs. 7B, C). GO enrichment analysis of these 116 intersecting genes revealed significant enrichment in cytokine regulation, DNA replication, and recombination pathways (Fig. 7D). KEGG pathway analysis further highlighted involvement of cytokine- and chemokine- related signaling pathways (Fig. 7E).

**Fig. 8:**
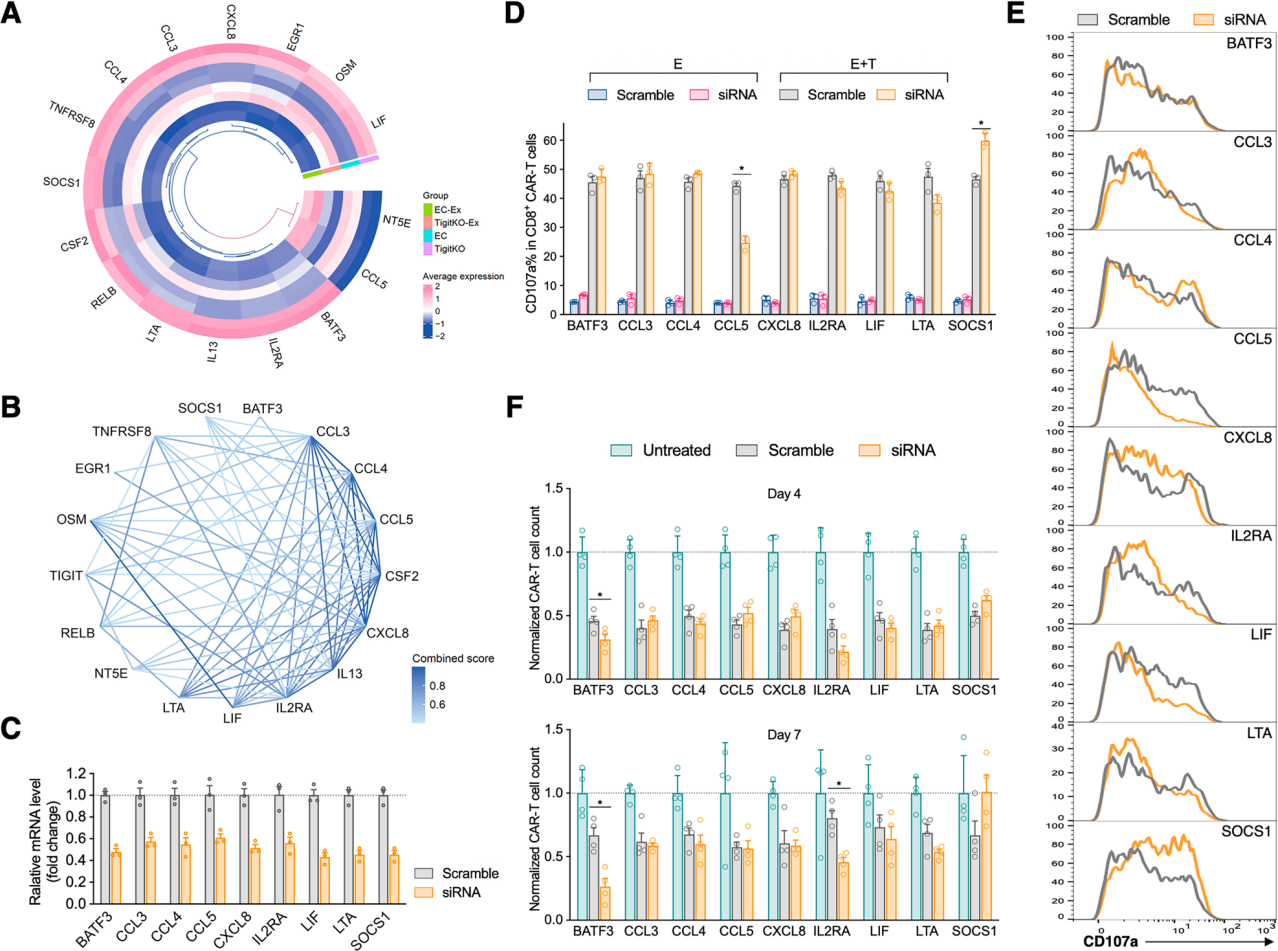
Preliminary functional validation of TIGIT downstream molecules. **A** Circular heatmap of expression differences for genes in the TIGIT-containing cluster. **B** PPI network of genes within the TIGIT-associated cluster. **C** Validation of knockdown efficiency for candidate genes by qPCR 48 h after siRNA transfection. **D** Effect of gene knockdown on CAR-T degranulation assessed by CD107a assay. **E** Representative plots of CAR-T degranulation after knockdown of different genes. **F** CAR-T cell counts on days 4 and 7 after siRNA-mediated gene knockdown (Data are presented as mean ± SEM; ^*^P < 0.05).

PPI analysis of these intersecting genes identified four distinct clusters, including a key cluster containing the TIGIT gene itself (highlighted in red, Fig. 7F). This cluster was rich in members of the cytokine family (e.g., IL13, IL2RA, CSF2), chemokine family (e.g., CCL3, CCL4, CCL5, CXCL18), and multiple molecules closely associated with T cell activation and exhaustion (e.g., BATF3, LTA, TNFRSF8, SOCS1). Expression analysis indicated that CCL5 and NT5E were downregulated following TIGIT knockout, whereas most other genes in the cluster were upregulated (Fig. 8A). A separate PPI analysis confirmed protein interactions between TIGIT and key genes in this cluster, including CCL4, CCL5, CSF2, IL2RA, TNFRSF8, and NT5E (Fig. 8B).

To functionally validate the impact of these TIGIT-regulated genes, we used siRNA to individually knock down several key genes (BATF3, CCL3, CCL4, CCL5, CXCL8, IL2RA, LIF, LTA, SOCS1) in CAR-T cells (Fig. 8C). A degranulation assay performed on day 4 post-knockdown revealed that CCL5 knockdown significantly impaired CAR-T cytotoxicity, whereas SOCS1 knockdown enhanced it (Fig. 8D, E). Cell counts on days 4 and 7 showed that BATF3 knockdown reduced CAR-T proliferation at both time points, while IL2RA knockdown increased cell numbers on day 7 (Fig. 8F). These results suggest that BATF3 is a key promoter of CAR-T proliferation, while CCL5 and SOCS1 act as critical regulators of CAR-T cytotoxic function.

## Discussion

Despite the remarkable efficacy of BCMA-CAR-T therapy for RRMM, relapse remains almost inevitable for most patients, posing significant challenges for subsequent treatment strategies. Thus, elucidating the mechanisms of resistance to CAR-T therapy and developing strategies to enhance and prolong the antitumor activity of CAR-T cells in vivo are of critical importance. Building on our previous research [8], we further analyzed T cell characteristics in patients receiving BCMA-CAR-T therapy to elucidate mechanisms underlying differential clinical outcomes. We found that DEGs between patients with early relapse and those with durable responses were significantly enriched in pathways related to T cell activation, proliferation, and differentiation. Notably, TIGIT expression in T cells was significantly higher in patients with early relapse.

Prior studies of CD19-CAR-T therapy for B cell lymphoma demonstrated elevated TIGIT expression in host T cells at relapse [20], and high TIGIT expression in CD19-CAR-T cells in vivo was linked to greater exhaustion and poorer prognosis [21]. Moreover, single-cell RNA sequencing has shown that exhausted CAR-T cells upregulate immune checkpoints such as LAG-3, TIM-3, CTLA-4, and TIGIT [22]. In our study, exposure of BCMA-CAR-T cells to target antigens (MM cells) induced time- and dose-dependent TIGIT upregulation, especially in the CD8^+^ T cells, accompanied by a loss of memory phenotype. Given the pivotal role of CD8⁺ T cells in mediating cytotoxic antitumor immunity, TIGIT upregulation attenuates their effector function and drives terminal exhaustion. This mechanism likely undermines CAR-T cell persistence and compromises therapeutic efficacy. Collectively, these findings highlight a close link between TIGIT expression and CAR-T cell exhaustion, suggesting TIGIT as a promising target for enhancing BCMA-CAR-T efficacy.

Although the clinical utility of immune checkpoint inhibitors (ICIs) is well established in solid tumors, conventional ICIs such as PD-1/PD-L1 and CTLA-4 inhibitors have shown limited efficacy in MM. TIGIT, first identified in 2009 [23], is a novel inhibitory receptor of the immunoglobulin superfamily [24, 25], primarily expressed on T cells and NK cells [24]. Aberrant TIGIT expression has been reported in tumor-infiltrating lymphocytes across various cancers, including gastrointestinal, breast, and squamous cell carcinoma [26]. Anti-TIGIT mAb have demonstrated clinical benefit in solid tumors in early-phase trials (NCT04524871 [27], NCT04540211, and NCT05329766). Recent studies also suggest an important role of TIGIT in MM, showing that both CD8^+^ T cells and Tregs exhibit high TIGIT expression, leading to impaired effector function and facilitating MM immune evasion [28]. Stromal cells and NK cells in the MM microenvironment further contribute to TIGIT-mediated suppression [29]. Preclinical evidence reinforced the therapeutic relevance of TIGIT in MM [30, 31]. The MyCheckpoint trial (NCT04150965) demonstrated that blockage of TIGIT and LAG-3 in heavily pretreated MM patients reshape the immune microenvironment and led to durable responses [32], while another trial (NCT04354246) is still ongoing. Consistent with these reports, our analysis revealed that high TIGIT in T cells, particularly CD8^+^ subsets, correlated with increased tumor burden and worse prognosis. Collectively, these findings suggest that targeting TIGIT may not only augment BCMA-CAR-T efficacy but also exert direct antimyeloma effects, positioning it as a promising target in the era of novel immunotherapies.

To dissect the role of TIGIT in BCMA-CAR-T therapy, we generated TIGIT-knockout CAR-T cells using CRISPR/Cas9 and applied anti-TIGIT mAb blockade. While TIGIT inhibition had limited impact on CAR-T function in vitro, in a humanized mouse model anti-TIGIT treatment markedly enhanced CAR-T expansion, reduced exhaustion, and improved antitumor efficacy. This discrepancy may stem from the absence of a complex immune microenvironment in vitro. Interestingly, anti-TIGIT mAb outperformed TIGIT knockout, likely because systemic antibody-mediated blockade modulates multiple immune subsets beyond CAR-T cells.

In a recent study on BCMA-CAR-T therapy, several TIGIT blockade strategies, including external anti-TIGIT mAb, a fourth-generation CAR-T design secreting a TIGIT-blocking scFv, and TIGIT knockout, enhanced CAR-T antitumor activity in vitro but yielded only modest survival benefits in vivo. Bioluminescence imaging in the external anti-TIGIT mAb method revealed no significant tumor reduction [16]. A key distinction in our study is the use of humanized mice reconstituted with PBMCs from the same donor as the CAR-T cells, enabling interactions between CAR-T cells, exogenous anti-TIGIT antibodies, and host immune components. Given the critical roles of host cells in microenvironmental remodeling and the bystander effects of cellular immunotherapy [33–35], this model provides a more physiologically relevant system. Under these conditions, exogenous anti-TIGIT mAb markedly enhanced BCMA-CAR-T efficacy, likely via supportive host interactions. Consistent with our findings, studies have shown that NK cells [36] and CD4^+^ CAR-T cell [37] upregulate TIGIT during CAR-T relapse, contributing to CAR-T dysfunction. Guan et al. [38] further demonstrated that anti-TIGIT mAb activates myeloid cells and drives CD8^+^ T cells from an exhausted effector state toward a memory-like phenotype. Together, these observations highlight the necessity of an intact immune network for realizing the full potential of checkpoint modulation in CAR-T therapy. In line with these observations, combined blockade of multiple checkpoints or strategies that boost host immunity may further potentiate CAR-T function [39], as supported by clinical evidence from combination checkpoint blockade trials [24].

While gene editing offers a powerful tool to modulate CAR-T function, our findings also highlight its limitations. CRISPR/Cas9-mediated TIGIT knockout may impair CAR-T fitness, as evidenced by faster tumor growth in the EC-CART group compared to the CART+IgG group. Considering the potential off-target effects associated with gene editing, antibody-mediated TIGIT blockade may represent a safer and more effective alternative. Notably, a recent study reported that bispecific antibody therapies in MM upregulate TIGIT and other immune checkpoints on T cells, thereby impairing T cell function and reducing antitumor efficacy [40]. Collectively, these data support the broader application of anti-TIGIT antibodies in combination with diverse immunotherapies for MM.

TIGIT plays a central role in a complex regulatory network involving multiple receptors (TIGIT, CD226, CD96, PVRIG) and ligands (CD155, CD112, CD111, CD113, Nectin-4), which together fine-tune the balance between stimulatory and inhibitory signaling [24]. We observed that CD96 expression was also elevated in MM T cells, particularly within the CD8^+^ subset, consistent with its overlapping inhibitory function with TIGIT. In contrast, CD226—an activating receptor that counteracts TIGIT-mediated suppression—showed no significant differences among the groups. CD155 (PVR), a major ligand in this axis, was highly expressed on MM cells [41] and bone marrow stromal cells [42], correlating with poor prognosis [41, 43]. Our histological analyses confirmed strong CD155 expression in the MM microenvironment. Notably, knockout of CD155 has been shown to enhance CAR-T cytotoxicity against MM [43], and recent preclinical data support CAR-T targeting CD155 in MM and other malignancies [44]. Moreover, comparative studies have demonstrated that anti-TIGIT antibodies with higher CD155-binding affinity exhibit superior antitumor activity [44]. Given the specific overexpression of CD155 in the MM microenvironment, targeting the TIGIT-CD155 axis represents an attractive strategy to overcome immunosuppression and CAR-T cell exhaustion.

To further elucidate the mechanisms underlying TIGIT blockade, we performed RNA sequencing on TigitKO CAR-T cells. TIGIT knockout altered the expression of multiple cytokines, chemokines, and T cell activation-related molecules, including CCL3, CCL4, LTA, and TNFRSF8 (CD30). Notably, TIGIT knockout upregulate BATF3, a transcription factor previously reported to mitigate T cell exhaustion and enhance CAR-T antitumor efficacy [45]. Our preliminary functional assays confirmed that BATF3 promotes the proliferation of BCMA-CAR-T cells, suggesting that this pathway contributes to the improved CAR-T performance observed with TIGIT inhibition. Further investigations, particularly in vivo studies, are warranted to fully elucidate the molecular mechanisms through which TIGIT regulates CAR-T exhaustion and functionality.

## Conclusion

In summary, while BCMA-CAR-T therapy represents a major advance in RRMM treatment, its durability remains limited, highlighting the urgent need for strategies that enhance efficacy. Through single-cell RNA sequencing and flow cytometry, we identified TIGIT overexpression as a key factor associated with relapse following BCMA-CAR-T therapy. Functional studies demonstrated that TIGIT blockade alleviates T cell exhaustion and enhances CAR-T antitumor activity, with anti-TIGIT mAb showing superior effects compared to TIGIT knockout. Mechanistically, RNA sequencing and preliminary functional experiments suggested that upregulation of BATF3 may be a key mechanism through which TIGIT regulates CAR-T cell function. These findings provide experimental evidence supporting TIGIT as a critical resistance mechanism in BCMA-CAR-T therapy and establish it as a promising therapeutic target in MM. Given the ongoing clinical development and anticipated availability of anti-TIGIT antibodies, combining BCMA-CAR-T therapy with TIGIT blockade may represent a promising strategy to improve clinical outcomes for MM patients.

## Supplementary information

Supplementary Table Legends

Supplementary Table 1

Supplementary Table 2

Supplementary Table 3

Supplementary Table 4

Supplementary Table 5

## Data Availability

The data supporting the conclusions of this research are available from the corresponding author upon reasonable request.
