# Supplementary material for: Preclinical evaluation of TIGIT as a target to enhance efficacy and mitigate T cell exhaustion in multiple myeloma following BCMA-CAR-T therapy
Source: Cell Death Dis. 2025 Dec 19;16(1):890. doi: 10.1038/s41419-025-08203-w (PMC12717268; doi:10.1038/s41419-025-08203-w)
Supplement: Supplementary file 1 — Supplementary Table Legends [file 41419_2025_8203_MOESM1_ESM.docx]

**SUPPLEMENTARY TABLE LEGENDS**

**Supplementary Table 1.** siRNA sequences used for gene knockdown.

**Supplementary Table 2.** Antibodies used for flow cytometry.

**Supplementary Table 3.** Primers used for PCR and qPCR.

**Supplementary Table 4.** RNA sequencing quality control and expression characteristics: (a) data quality control; (b) mapping statistics of reads to reference; (c) distribution of mapped reads across genomic regions, (d) distribution statistics of gene expression levels in samples, (e) distribution statistics of different gene expression intervals in samples, (f) Statistics of the transcript expression coverage depth.

**Supplementary Table 5.** DEGs between durable response (DR) and early relapse (ER) groups, along with pan-cancer immune regulatory gene profiles.
